# Searching for the ground state of complex spin-ice systems using deep learning techniques

**DOI:** 10.1038/s41598-022-19312-3

**Published:** 2022-09-02

**Authors:** H. Y. Kwon, H. G. Yoon, S. M. Park, D. B. Lee, D. Shi, Y. Z. Wu, J. W. Choi, C. Won

**Affiliations:** 1 Center for Spintronics, Korea Institute of Science and Technology, Seoul, 02792 South Korea; 2 Department of Physics, Kyung Hee University, Seoul, 02447 South Korea; 3 School of Physical Science and Technology, ShanghaiTech University, Shanghai, 201210 China; 4 Department of Physics, State Key Laboratory of Surface Physics, Fudan University, Shanghai, 200433 China; 5 Shanghai Research Center for Quantum Sciences, Shanghai, 201315 China

**Keywords:** Ferromagnetism, Magnetic properties and materials, Information theory and computation

## Abstract

Searching for the ground state of a given system is one of the most fundamental and classical questions in scientific research fields. However, when the system is complex and large, it often becomes an intractable problem; there is essentially no possibility of finding a global energy minimum state with reasonable computational resources. Recently, a novel method based on deep learning techniques was devised as an innovative optimization method to estimate the ground state. We apply this method to one of the most complicated spin-ice systems, aperiodic Penrose P3 patterns. From the results, we discover new configurations of topologically induced emergent frustrated spins, different from those previously known. Additionally, a candidate of the ground state for a still unexplored type of Penrose P3 spin-ice system is first proposed through this study. We anticipate that the capabilities of the deep learning techniques will not only improve our understanding on the physical properties of artificial spin-ice systems, but also bring about significant advances in a wide range of scientific research fields requiring computational approaches for optimization.

## Introduction

Searching for the ground state, the lowest energy state of a system, is one of the most important problems in a wide range of scientific research fields. However, the only way to definitively determine the true ground state is by scanning all the possible states and comparing their energies, and hence, the difficulty of this problem increases exponentially as the system size increases. Various computational approaches, such as the simulated annealing implemented by the Monte-Carlo method, are utilized to estimate the ground state of a system. Yet, only local energy minimum states close to the ground state, rather than the exact ground state, can be reasonably obtained using the conventional methods.

Given the dire situation, researchers in various research fields have great hope toward deep learning techniques. These computational techniques make it possible for computers to solve problems without being explicitly programmed to do so, thus they have been extensively adopted to solve complex problems in scientific research^1–3^. Indeed, the deep learning techniques have also been utilized in a classical topic of condensed matter physics, searching for the ground state of a system^4–8^. Several previous studies have been performed to show the possibilities that deep learning techniques can be used to enhance conventional methods by reducing computational costs^9,10^ and to devise novel simulation methods generating various energetically stable physical states^11–13^. However, these methods still do not include the process for effectively reducing the total degrees of freedom in the system, and hence, the difficulty of searching for the true ground state cannot be significantly reduced even with the methods.

Recently, an innovative ground state estimation method based on a novel deep generative model, Energy-minimization variational autoencoder (E-VAE), was devised^14^. The E-VAE model is composed of the encoder and decoder network structures similar to the original variational autoencoder (VAE) model^15^, and the encoder part compresses input data into a new representation in the reduced data dimension. It is expected that the compression process can reduce the search area of the possible solutions, thus the problems that were unreachable with traditional methods can be reasonably handled with the available computational resource. Additionally, the Hamiltonian of a given system is considered explicitly in the training process of the E-VAE model, so that it can learn not only revealed features but also unrevealed physics in the training dataset. This implies that, even though the dataset does not include the ground state of a given system, it is possible to generate the ground state using a well-trained E-VAE model. As suggested in a previous study^14^, to obtain a spin state close to the ground state of the system, we can deliberately utilize a collapsing phenomenon of the E-VAE model by increasing the influence of the Hamiltonian considered in the training process.

In this study, we apply the E-VAE model to search for the ground states of complex dipolar artificial spin-ice systems. Artificial spin-ice systems are typically composed of interacting magnetic dipole moments located on the frame structures designed to generate frustrated spin systems. These systems have been studied intensively due to their interesting physical properties including emergent magnetic monopoles^16–18^, vertex-based frustration^19,20^, and thermal excitations^21,22^. A representative research objective in the field of artificial spin-ice systems is to search for the highly-degenerate ground states induced by the geometrical frustration of the systems^23–25^. However, it is known that the probability of obtaining the ground state of artificial spin-ice systems usually decreases dramatically as the size of the system increases. As an example, in the case of the artificial Kagome spin-ice system, as the number of building block structures increases there is a dramatic decrease in the ability to achieve the ground state^26–28^. In the cases of artificial magnetic quasicrystal spin-ice systems with Penrose patterns^29–32^, obtaining the ground states of the systems becomes a more challenging problem; a locally low-energy spin configuration in a small system cannot be generally extended to the ground state of a larger system, owing to the lack of translational symmetry in the aperiodic patterns^33^. Here, we construct two types of different Penrose P3 patterned dipolar spin-ice systems, Type-I and -II, that we apply our E-VAE model to.

The Type-I system has been studied experimentally by Shi et al.^34^ through a real-space implementation of the system with many narrow magnetic islands. They proposed a ground state candidate of the system, composed of a quasi-one-dimensional rigid part (skeleton part) and the topologically induced emergent frustration part (flippable part). We apply the E-VAE model to confirm whether the proposed candidate of the ground state is the lowest energy state of the system. Through a detailed analysis, we reveal that the skeleton and flippable configurations shown in our result are not exactly the same as the one proposed by Shi et al. in the previous study^34^. This discovery implies that the problem to find a global rule determining the frustrated parts, is not entirely solved. In this study, we propose a new skeleton and flippable configuration.

The Type-II Penrose P3 spin-ice system, which has a different network frame structure compared with Type-I system, has never been clarified before to the best of our knowledge. We apply the E-VAE model to search for the uncharted ground state of the Type-II system, and obtain a spin state which has a significantly lower energy value compared with those found using a conventional simulated annealing method^29,35^. In addition, we find a general characteristic of Penrose P3 spin-ice system, regardless of the type of patterns, in that there are topologically induced emergent frustration parts.

## Strategy

### Network structure

**Figure 1:**
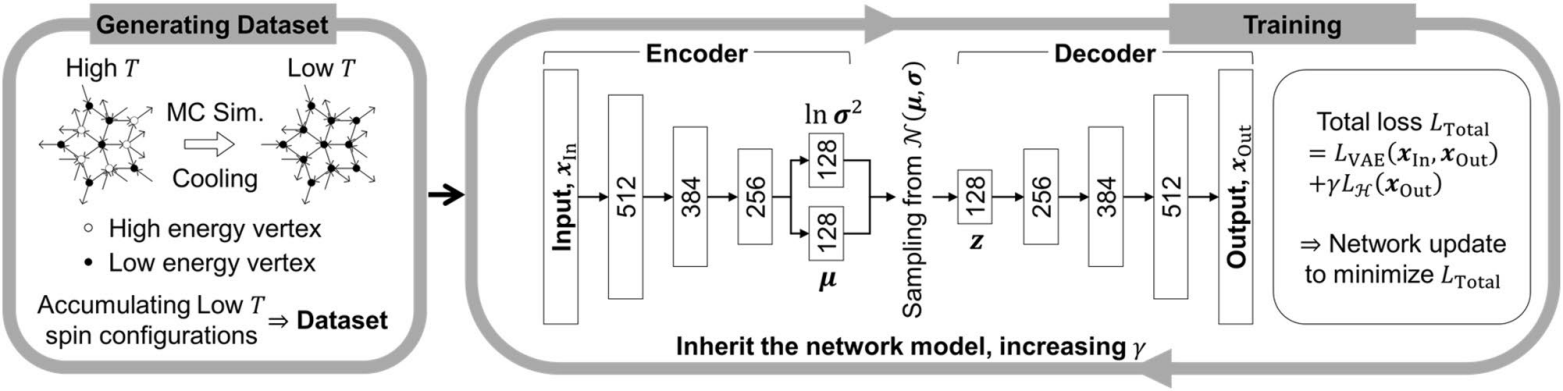
Deep learning process to search for the ground states of complex spin-ice systems. A schematic diagram for the dataset generation and the training process of E-VAE model used in this study. Simulated annealing implemented by a Monte-Carlo simulation (MC Sim.) is used to generate spin configuration datasets. Fully-connected neural network layers are used to implement the network structure, and the numbers indicate the hidden units of each layers. See “Methods” section for a detailed explanation.

To search for the spin states close to the ground state of spin-ice systems considered in this study, we implement an E-VAE model using the neural network structure shown in Fig. 1. The detailed information for the network structure is given in the “Methods” section. Here, we focus on the modifications made to fit the E-VAE network structure to the spin-ice systems.

First, we represent the input data as a series of binarized numbers (− 1 or 1). We numerically implement several spin-ice systems using point dipoles, and generate numerous metastable spin states using a conventional simulated annealing method^29^ on each system. More discussion about the generated data, specific to each spin system, is given in the “Results” section. Each data is composed of $N$ dipoles, and each dipole is considered to behave like an Ising spin; it has a normalized magnetic moment that can only flip, but not rotate. Thus, input data can be transformed into an $N$-dimensional vector including $N$ binarized numbers, and each number implies one of the two directions for each dipole.

Second, the output data is composed of $N$ numbers in the range of − 1 to 1; there is no normalization process that forces each component of output data to be exactly − 1 or 1. The training process of typical neural network models uses a differentiation process to update their network parameters, which is called the back-propagation process^36^. Therefore, there should be at least one differentiable variable to train the network properly. For that reason, we do not apply the normalization process in our network since the magnitude is the only continuous and differentiable variable to determine each component of the output data through our network structure. Instead, the hyperbolic tangent function is used as the activation function of the output layer, mapping each component of the output data between − 1 to 1. We consider each component of the output data as indicator of the certainty regarding the direction of each Ising-like spin.

### Loss function and training process

In a typical training process of a deep learning algorithm, the neural network is updated to minimize a loss function consisting of multiple loss terms, with each minimization of the loss term representing a distinct training goal. Likewise, the E-VAE model is trained to minimize the total loss function $L_{{{\text{Total}}}}$ consisting of the conventional VAE loss $L_{{{\text{VAE}}}}$ and the Hamiltonian loss $L_{{\mathcal{H}}}$: $L_{{{\text{Total}}}} = L_{{{\text{VAE}}}} + \gamma L_{{\mathcal{H}}}$^14^, where $\gamma$ is the coefficient of $L_{{\mathcal{H}}}$. The $L_{{{\text{VAE}}}}$ is composed of two losses, the reconstruction loss $L_{{{\text{RC}}}}$ and the Kullback–Leibler (KL) loss $L_{{{\text{KL}}}}$. The mathematical expressions and the training objectives of these terms in the E-VAE model are given in the “Methods” section and previous studies^14,15,37^. The Hamiltonian loss term, $L_{{\mathcal{H}}}$, is related to the physics of the target system. In this study, we consider the dipole–dipole interaction between the magnetic moments in the spin-ice systems, thus, the Hamiltonian loss term becomes the dipole–dipole interaction energy calculated from the output data of the E-VAE model.

Increasing $\gamma$ during the training process of E-VAE model makes the spin states from the trained model energetically more stable^14^. In particular, when $\gamma$ exceeds a certain value, the trained E-VAE model collapses drastically and the output states converge into a single state. The state has the lowest energy value of all the states that the trained E-VAE network produces, and hence, is possibly the ground state of the system. Utilizing this feature of the collapsed E-VAE model, we initially start a training process of an E-VAE model with $\gamma = 0$ condition and gradually increase to $\gamma = 5$ so that the model can successfully approach the ground state of spin-ice systems (see the “Methods” section for more details).

## Results

### Type-I Penrose P3 system

#### Target system

**Figure 2:**
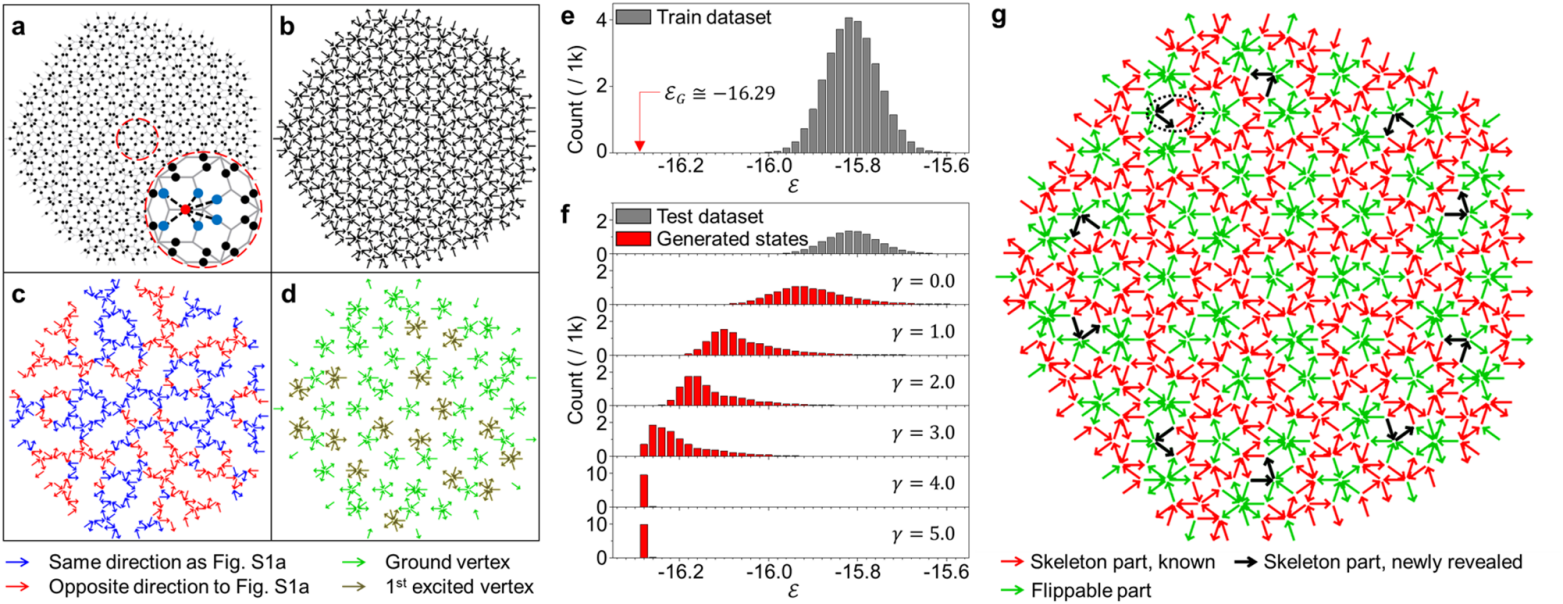
Structure and generated spin states of the Type-I Penrose P3 system. (**a**) The frame structure of Type-I system composed of 805 spins. Each circular dot represents where each spin is located. The sub-figure shows the dipole–dipole interaction scheme considered in this system, where blue dots indicate the spins interacting with the one on the red dot. (**b**) A sample spin state in the test dataset. (**c, d**) Spins of the spin state (**b**) that are located at the skeleton (**c**) and flippable (**d**) parts. (**e**) $\varepsilon$ distribution for the training dataset. $\varepsilon_{G}$ indicates the energy density value of the proposed ground state. (**f**) $\varepsilon$ distributions of the test dataset and the generated states from the trained E-VAE model with each $\gamma$ value. (**g**) The lowest energy spin state obtained from a trained E-VAE model with $\gamma = 5.0$. The black dotted circle represents the clockwise flow formed by the black and red spins.

One of the main objectives of this study is to confirm that the E-VAE model can be used as an efficient computational approach to search for energetically stabilized spin states on a complicated spin-ice system. For this, we implement a Penrose P3 dipolar spin-ice system which is referred to as a Type-I system in this study (Fig. 2a).

This system has a quasi-crystalline pattern composed of seven different types of unit vertices^34^; Each magnetic moment is centered on the side of two types of rhombuses composed of Penrose tiles, and its direction is strongly constrained to be parallel with the side. Translational symmetry is not available on this system^33^, thus the difficulty of searching for the true ground state increases exponentially with the size of the system. Nevertheless, Shi et al. have proposed a method to build up the ground state for this system through elaborated logical steps^34^ (see Fig. S1 in Supplementary Information, SI). They found that the proposed ground state consists of two different spin groups, the skeleton and flippable parts. The skeleton part is a quasi-one-dimensional rigid part, and it has a unique (up to time-reversal symmetry) ground state with long-range ordering. The flippable part is composed of topologically induced emergent frustrated vertices or spins^20,34^. These are usually surrounded by the skeleton part, and they can be flipped to other degenerate spin configurations with identical energy. To demonstrate the utility of E-VAE model in searching for the ground states of spin-ice systems, we set this Type-I system as our first target system and investigate whether the E-VAE model can generate the proposed ground state properly.

As shown in the sub-figure of Fig. 2a, the dipole–dipole interaction model is considered only between the nearest neighbors as indicated by Eq. (1),

1 $$\varepsilon = \frac{E}{ND} = - \frac{1}{N}\mathop \sum \limits_{ij} \frac{{3\left( {\hat{m}_{i} \cdot \vec{r}_{ij} } \right)\left( {\hat{m}_{j} \cdot \vec{r}_{ij} } \right) - \hat{m}_{i} \cdot \hat{m}_{j} \left| {\vec{r}_{ij} } \right|^{2} }}{{\left| {\vec{r}_{ij} } \right|^{5} }},$$

where $\varepsilon$ is a unitless energy density parameter, $E$ is the total energy of the system, $D$ is the dipole–dipole interaction strength written in energy unit, $\hat{m}_{i}$ is a magnetic moment located at ith spin site, and $\vec{r}_{ij}$ is the unitless displacement vector between the dipole moments $\hat{m}_{i}$ and $\hat{m}_{j}$. The $ij$ index pair under the summation refers to the nearest neighbor pairs. This short-range interaction scheme is adopted to exactly follow the logical steps that Shi et al. used to obtain the ground state by tiling vertices.

#### Dataset generation

To secure the dataset composed of numerous metastable spin states, we independently perform multiple simulated annealing processes on the system and gather the final local energy minimum states. The total dataset is composed of 50,000 spin states, and it is divided into three sub-datasets: training, validation, and test datasets with 30,000, 10,000, and 10,000 data, respectively. The details about the simulated annealing process and data generation are given in the “Methods” section.

A sample of the simulated annealing results is shown in Fig. 2b. Here, we first show that simulated annealing is not an appropriate method to search for the ground state of complex spin-ice systems. To demonstrate this clearly, we separated the spins in this sample into two groups which are composed of the spins located on the skeleton (Fig. 2c) and flippable (Fig. 2d) parts of the proposed ground state (Fig. S1a in SI). It is clearly shown that the two colors (red and blue) form several domains, and the domains are intermixed in most regions in Fig. 2c. Considering that the magnetic moments in the skeleton part in the proposed ground state make a specific long-range ordering, the intermixed domains indicate that the simulated annealing method which is one of the representative conventional optimization methods is not enough to investigate the true ground state due to the enormous complexity and the existence of numerous metastable states of this system. In addition, there are lots of vertices in their excited states as shown in Fig. 2d, and it clearly indicates that the spin state shown in Fig. 2b is not in the lowest energy state.

We investigated the energy density histogram for the 30,000 spin states in our training dataset (simulated annealing results) as shown in Fig. 2e. The $\varepsilon_{G}$, which is the energy value calculated using the Eq. (1) and the spin configuration of the proposed ground state by Shi et al., is significantly lower than the $\varepsilon$ values of the histogram. This fact qualitatively supports that the simulated annealing method has limitations for solving this complex spin-ice system.

#### Training results

We train our E-VAE model using the metastable spin states in the training dataset and investigate the energy distributions of generated states from the trained E-VAE models for each $\gamma$ value as shown in Fig. 2f. As $\gamma$ increases, the energy distribution shifts to a lower energy region. Considering the Hamiltonian loss, $L_{{\mathcal{H}}}$, is related to the energy of the generated state and $\gamma$ is the controlling parameter for the independent variation of the magnitude of ${ }L_{{\mathcal{H}}}$, the behavior of energy distribution shown in Fig. 2f indicates that the $L_{{\mathcal{H}}}$ is properly minimized during the training process of E-VAE model. (The behaviors of each loss term during the training process are given in Fig. S2 and Note 1 in SI) In addition, the energy distribution collapses into a sharp peak when $\gamma$ exceeds a certain value; in Fig. 2f, it is approximately 3.0–4.0. In other words, the collapsed E-VAE model generates a sufficiently lower energy spin state in the Type-I system compared with the simulated annealing results. Hence, we confirm that the E-VAE model can be utilized as an innovative numerical method significantly outperforming conventional methods in estimating the ground state of the complex spin-ice system.

The generated spin state from the collapsed E-VAE model trained with $\gamma = 5.0$ is shown in Fig. 2g. Comparing with the skeleton and flippable parts of the simulated annealing result shown in Fig. 2c, d, there is a perfectly ordered skeleton structure with the fivefold rotational symmetry which is implied in the frame structure of the system, and all vertices in the flippable part are in one of their degenerate ground state configurations. Thus, the state found by the E-VAE model is one of the possible configurations of the spin state proposed by Shi et al. ($\varepsilon$ of the generated spin state is exactly the same as $\varepsilon_{G}$), clearly supporting that it is a candidate for the lowest energy state of the Type-I system.

In addition, the E-VAE reveals a surprising fact: the spins highlighted as the black arrows in Fig. 2g at several specific five-fold vertices, though known as representative flippable vertices^34^, should actually be classified as the skeleton part, and not the flippable part. One can notice that there is an anti-clockwise flow formed by the black and red spins, as indicated by the black dotted circle in Fig. 2g, which can be found repeatedly throughout the spin states at the specific five-fold vortex sites. If the five-fold vertices are truly independent flippable vertices, then the anti-clockwise and clockwise flows of black arrows should appear randomly. Thus, we suspect that the spins highlighted as the black arrows are not flippable but rigid. All possible configurations are checked for the rigidity of these sites (see Fig. S3 in SI), and we confirm that the spins cannot be flipped until the entire skeleton structure is inversed. Consequently, we propose a new skeleton-flippable configuration of ground state including the newly revealed additional skeleton parts as shown in Fig. 2g. It displays accurately which spins are truly flippable when the system reaches its lowest energy state.

Observing these newly revealed additional skeleton parts, one can notice that the degeneracy of the flippable parts is greatly reduced. In other words, not all the spin configurations that are previously proposed as the ground states are the ground state. For each of the specific five-fold vertices including the black arrows, only four configurations are degenerate as the ground state. This is less than half of the previously proposed ten-fold degeneracy (five-fold and time-reversal symmetry) as shown in Fig. S1b in SI. The six excluded configurations have higher energies than the four ground state configurations. Specifically, in these excluded configurations, a flippable vertex (VII-type vertices shown in Fig. S1b) neighboring the five-fold vertices should be in the first excited state, not in the ground state. The energy difference between the excluded states and the true ground state is $0.9653 \times \frac{{\mu_{0} m^{2} }}{{4\pi a^{3} }}$, where $\mu_{0}$ is vacuum permeability, $m$ is the magnitude of a magnetic moment, and $a$ is intervertex spacing. The value is estimated to be about 14 meV using the experimental condition in a previous study^34^, as $m = M_{{\text{s}}} V$, $M_{{\text{s}}} = 800\;{\text{kA}}/{\text{m}}$, $V = 6.67 \times 10^{ - 22} \;{\text{m}}^{3}$, $a = 500{ }\;{\text{nm}}$. $M_{{\text{s}}}$ is the saturation magnetization of permalloy at room temperature and $V$ is the volume of a magnetic island used in the experiment.

The ground state configuration proposed using our method has not been experimentally realized yet; so far, the experimentally observed states have much higher energy that our proposed ground state configuration. More ordered states can be approached as the coupling strength between the spins increase. Nevertheless, a previous experimental study^34^ shows that this target system can only reasonably reach locally stable states with a multi-domain structure, and not its true ground state, due to the complex and aperiodic pattern of the Penrose P3 tiling. Further experimental studies are required to examine how further ordering can occur to access the true ground state of this system.

### Type-II Penrose P3 system

#### Target system

**Figure 3:**
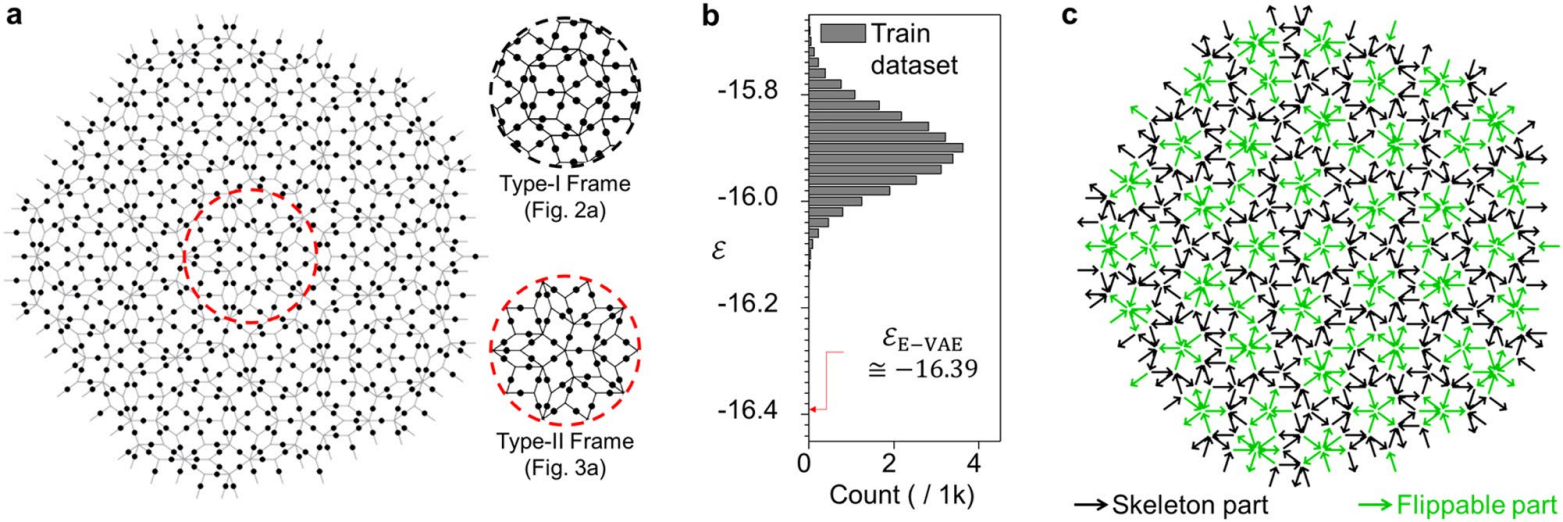
Structure and generated spin states of the Type-II Penrose P3 system. (**a**) The frame structure of Type-II system composed of 640 spins. Each circular dot represents where each spin is located. Two sub-figures show the magnified views around the center of Type-I and -II frame structures. (**b**) $\varepsilon$ distribution for the training dataset. (**c**) The lowest energy spin state obtained from a trained E-VAE model with $\gamma = 5.0$ in Type-II system. $\varepsilon_{{{\text{E}} - {\text{VAE}}}}$ in (b) indicates the energy density value of the spin state shown in (c).

To search for the ground states of unexplored spin-ice systems, we use a different type of aperiodic Penrose P3 pattern to implement complicated spin-ice systems which are referred to as Type-II systems in this study. We construct three Type-II systems including 640, 1195, and 2150 spins; Fig. 3a shows the frame structure of a Type-II system with 640 spins. Note that the frame structure of the system is clearly distinct from the Type-I system as shown in the sub-figures in Fig. 3a. The ground states of Type-II systems have never been investigated before to the best of our knowledge. The short-range interaction scheme shown in Eq. (1) is also considered in Type-II systems to generate datasets and to define the Hamiltonian losses for each system.

#### Proposing the ground state

We first apply the E-VAE model to the Type-II system with $N = 640$. The same network structure shown in Fig. 1 is also used to implement the E-VAE model for this case. We generate datasets and train the E-VAE model using the same strategy as for the Type-I system.

After the training process, we obtain a spin state from the trained E-VAE model. The energy value of the spin state is significantly lower than the values in the energy distribution of the simulated-annealing-generated spin states (Fig. 3b). Figure 3c shows the spin state obtained from the trained E-VAE model, and we propose it as a candidate of the ground state of the Type-II system. Similar to the Type-I system, the Type-II system also shows a robust skeleton part, although it has a different quasi one-dimensional ordering. We also confirm the existence of the flippable part: each vertex can be replaced by a different spin configuration with identical energy. Although only applied to two different spin-ice systems, we believe our E-VAE model has the potential to be utilized to estimate the ground state of various complex spin systems.

#### System size dependency

As discussed earlier, the difficulty of determining the ground state of the Penrose P3 spin-ice systems using conventional methods increases exponentially with the system size, owing to the system being composed of aperiodic patterns with no translational symmetry in the spin network structures. On the contrary, we speculate that the difficulty of searching for the ground state using the E-VAE model may not be greatly affected by the size of the system owing to an efficient grouping process of the encoder network structure.

The E-VAE model is designed to include an encoder and decoder structure as shown in Fig. 1. The encoder network compresses the given input data into a single latent code. Through this encoding process, it is expected that several essential features from the vast amount of information implied in the input data are extracted and encoded into the latent code. Therefore, each component in the code represents collective characteristics (usually called high-level features in the deep learning field), combining several simple characteristics of the input data. In the case of the spin-ice systems considered in this study, each component in the latent codes can be connected to various collective states composed of multiple spins. Especially, if there is a long-range ordered structure in the ground state of the target system, such as the spins in the skeleton parts which has two degenerate time-reversed configurations, it is expected that the total information of the multiple-spin-ordered-structure can be encoded into a few components of the latent code. In other words, the encoder part in the E-VAE effectively reduces the total degree of freedom of the raw data through an efficient grouping process.

**Figure 4:**
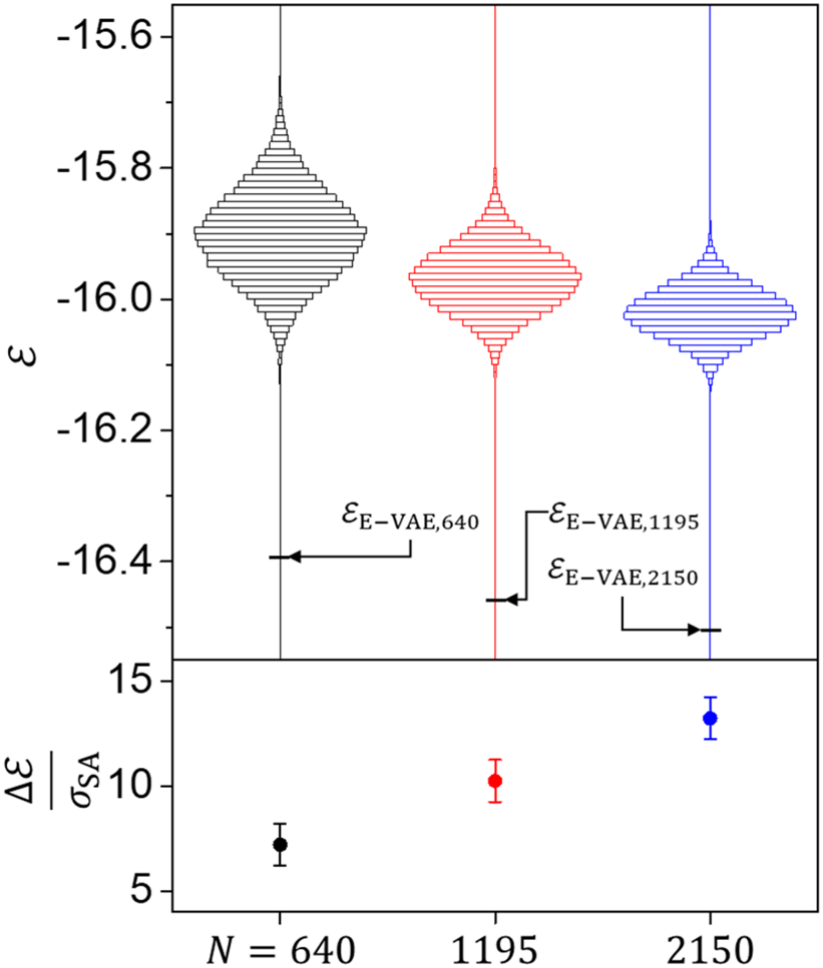
Estimating the ground states using E-VAE for systems of different sizes. Comparison between results of simulated annealing and our method using the E-VAE model for the $N = 640$, 1195, and 2150 cases. $\varepsilon$ histograms are calculated using the training datasets, and $\varepsilon_{{{\text{E}} - {\text{VAE}},N}}$ s indicate the energy density values of the spin states obtained from the trained E-VAE models for each of the $N$ cases. $\Delta \varepsilon /\sigma_{{{\text{SA}}}}$ represents $\left( {\mu_{{{\text{SA}}}} - \varepsilon_{{{\text{E}} - {\text{VAE}}}} } \right)/\sigma_{{{\text{SA}}}}$, where $\mu_{{{\text{SA}}}}$ and $\sigma_{{{\text{SA}}}}$ are the mean and standard deviation values of $\varepsilon$ histograms.

In order to validate this claim, we apply E-VAE model to two other Type-II systems with different $N$ ($N = 1195$ and 2150). The spin states found by the trained E-VAE models for each of these $N$ cases are shown in Fig. S4 in SI, while the ground state energies are shown in Fig. 4.

For all cases, the trained E-VAE model discovers a significantly lower energy state than the energy distribution of the simulated-annealing-generated dataset used to train each model. Considering that standard deviation value of each distribution, $\sigma_{{{\text{SA}}}}$, represents the energy difference between the spin states that can be reasonably obtained through the simulated annealing process, it is surprising that the energy value of each spin state obtained from the trained E-VAE model is several times of $\sigma_{{{\text{SA}}}}$ away from the mean value of each distribution, $\mu_{{{\text{SA}}}}$.

To quantitatively show the remarkable performance of our method, we investigate the $\Delta \varepsilon /\sigma_{{{\text{SA}}}}$ which is a measure of the energy difference between $\varepsilon_{{{\text{E}} - {\text{VAE}}}}$ and $\mu_{{{\text{SA}}}}$ ($\Delta \varepsilon /\sigma_{{{\text{SA}}}}$ graph in Fig. 4). Note that $\Delta \varepsilon /\sigma_{{{\text{SA}}}}$ increases with the system size ($N$). For instance, in the case of $N = 2150$, $\Delta \varepsilon$ is as large as $13\sigma_{{{\text{SA}}}}$. Following empirical rule, the probability ($P$) of obtaining a spin state with the energy value of $\varepsilon_{{{\text{E}} - {\text{VAE}}}}$ through the simulated annealing process is infinitesimal ($P \approx 10^{ - 38}$ for $13\sigma_{{{\text{SA}}}}$). Therefore, our E-VAE method significantly outperforms the simulated annealing method; this advantage becomes more prominent when the system size increases.

Our method shares general limitations and drawbacks existing in common deep learning algorithms, such as the requirement of a big dataset and difficulty in explaining how the trained machine obtains the solution^38^. Nevertheless, we believe that this method can transcend the performance of conventional methods in the problems of searching for the ground state of various spin-ice systems, particularly those with novel complex tiling patterns, intractable interaction terms, and large sizes.

## Conclusion

We adopted a novel optimization method based on the E-VAE model, which is a deep generative model, to study complicated spin-ice systems. We confirmed that the E-VAE model can be used as an efficient computational approach to find energetically minimized spin states of two distinct types of aperiodic Penrose P3 patterns. As a result, we discovered for the Type-I Penrose P3 spin-ice system that topologically induced emergent frustration part (flippable part) appears in a modified configuration from the previously known one, and we presented the new skeleton-flippable configuration including the additionally revealed skeleton parts. Moreover, we proposed a candidate of the ground state of a Type-II Penrose P3 spin-ice system which has never been clarified before, and it is confirmed that there are topologically induced emergent frustration parts regardless of the types of Penrose P3 patterns. In addition, we found that our method can efficiently estimate the ground states even when the size of the spin-system increases, up to sizes that would render conventional optimization method nearly useless.

Our work shows that the E-VAE model transcends the limitations of the conventional optimization methods in searching for ground state problems. We believe that our method can be generalized and applied to explore various complex systems, thereby leading to a broad and deep impact in various research disciplines.

## Methods

### Structural information of network structure

As shown in Fig. 1, the encoder part consists of four fully-connected neural network layers with 512, 384, 256, and 256 (128 + 128) hidden units, and the last 256 components are divided into two groups to be used as the mean (${\varvec{\mu}}$) and log-variance ($\ln {\varvec{\sigma}}^{2}$) values. A Batch-Normalization (BN) and a leaky-ReLu activation layer are attached after each neural network layer except the last neural network layer in the Encoder part. After the Encoder part, ${\varvec{z}}$, which is usually referred as a latent code, is sampled from a set of normal distributions constructed using the ${\varvec{\mu}}$ and ${\varvec{\sigma}}$. Lastly, in the decoder part, ${\varvec{z}}$ is decoded into an output data composed of $N$ numbers through four fully-connected neural network layers with 256, 384, 512, and $N$ hidden units. A BN and a leaky-ReLu activation are also attached after each neural network except the last layer. For the last neural network layer in the decoder part, a tanh activation is used to ensure that all components of an output data are in the range of − 1 to 1. The Adam optimizer^39^ was employed to train this network structure. The learning rate, $\beta_{1}$, and $\beta_{2}$ which are the hyper-parameters of the Adam optimizer are fixed at 0.001, 0.9, and 0.999, respectively.

### E-VAE loss function

As introduced in a previous study^14^, the total loss function of the E-VAE model, $L_{{{\text{Total}}}}$, is composed of conventional VAE losses and the Hamiltonian loss as shown in Eq. (2),

2 $$\begin{aligned} & L_{{{\text{Total}}}} = L_{{{\text{RC}}}} + \beta L_{{{\text{KL}}}} + \gamma L_{{\mathcal{H}}} , \\ & L_{{{\text{RC}}}} = \left( {m_{{{\text{In}},i}} - m_{{{\text{Out}},i}} } \right)^{2}_{i} , \\ & L_{{{\text{KL}}}} = \frac{1}{2}\mathop \sum \limits_{n}^{128} \left( {\sigma_{n}^{2} + \mu_{n}^{2} - \ln \sigma_{n}^{2} - 1} \right), \\ & L_{{\mathcal{H}}} = \frac{{E_{{{\text{Out}}}} }}{ND} = - \frac{1}{N}\mathop \sum \limits_{ij} \frac{{3\left( {\vec{m}_{{{\text{Out}},i}} \cdot \vec{r}_{ij} } \right)\left( {\vec{m}_{{{\text{Out}},j}} \cdot \vec{r}_{ij} } \right) - \vec{m}_{{{\text{Out}},i}} \cdot \vec{m}_{{{\text{Out}},j}} \left| {\vec{r}_{ij} } \right|^{2} }}{{\left| {\vec{r}_{ij} } \right|^{5} }}. \\ \end{aligned}$$

The $L_{{{\text{Total}}}}$ is minimized during the training process of E-VAE model. The minimization of each loss term represents distinct training objectives. The goal of minimizing the reconstruction loss, $L_{{{\text{RC}}}}$, is to make output data identical to input data, where $m_{{{\text{In}},i}}$ and $m_{{{\text{Out}},i}}$ are the ith components of input and output data. The goal of minimizing the KL loss, $L_{{{\text{KL}}}}$, is to increase the similarity between a standard normal distribution and the feature distributions, which are the set of normal distributions constructed using the ${\varvec{\mu}}$ and ${\varvec{\sigma}}$ values; $L_{{{\text{KL}}}}$ is minimized when all $\mu_{n}$ and $\sigma_{n}$ are 0 and 1, respectively. $\beta$, the coefficient of $L_{{{\text{KL}}}}$ term, is set to be 0.005 with the appropriate rationales given in the Fig. S5 and Note 2. The goal of the Hamiltonian loss term, $L_{{\mathcal{H}}}$, is to minimize the dipole–dipole interaction energy calculated using the generated spin state (output data); the interaction scheme shown in Eq. (1) is used to calculate this $L_{{\mathcal{H}}}$ term, with only difference being that the magnetic moments in the Eq. (1) are replaced by the components of output data. The $\gamma$ is controlled during the training process of the E-VAE model.

### Training process

We initially start a training process of a E-VAE model with $\gamma = 0$ condition (same as the conventional $\beta$-VAE model^37^), and increase $\gamma$ every 200 training epochs with a 0.05 step size until it reaches 5. It is confirmed that the condition $\gamma = 5$ is sufficient to collapse the energy distributions of the spin states generated from all E-VAE models trained in this study. The batch size used in all training processes is 500.

### Simulated annealing process and data generation

We implement a simulated annealing process using the Metropolis–Hastings algorithm which is one of the representative Monte-Carlo methods^29^. During a simulated annealing process, the temperature of the system decreases linearly from a high enough temperature to zero temperature to obtain various metastable spin states; initial temperature is set to $T = 30$, confirming that the initial temperature is sufficient to make all systems considered in this study paramagnetic. We independently perform the simulated annealing process multiple times to generate 50,000 metastable spin states for each system. The total dataset is divided into three sub-datasets to train the network structure (30,000 data for training dataset), to monitor the training process (10,000 data for validation dataset), and to evaluate the performances of the trained network (10,000 data for test dataset). It is confirmed that there is no duplicate data (same spin states) in all total datasets.

## Supplementary Information

Supplementary Information.

## Data Availability

All data needed to evaluate the conclusions in the paper are present in the paper and/or the Supplementary Materials. Additional data related to this paper may be requested from H.Y.K.
