# Supplementary material for: Searching for the ground state of complex spin-ice systems using deep learning techniques
Source: Sci Rep. 2022 Sep 2;12:15026. doi: 10.1038/s41598-022-19312-3 (PMC9440018; doi:10.1038/s41598-022-19312-3)
Supplement: Supplementary file 1 — Supplementary Information. [file 41598_2022_19312_MOESM1_ESM.docx]

Supplementary Materials for

**Searching for the ground state of complex spin-ice systems using deep learning techniques**

H. Y. Kwon^1*^, H. G. Yoon^2^, S. M. Park^2^, D. B. Lee^2^, D. Shi^3^, Y. Z. Wu^4,5^, J. W. Choi^1^, and C. Won^2*^

^1^**Center for Spintronics, Korea Institute of Science and Technology, Seoul 02792, South Korea**

^2^**Department of Physics, Kyung Hee University, Seoul 02447, South Korea**

^3^**School of Physical Science and Technology, ShanghaiTech University, Shanghai 201210, China**

^4^**Department of Physics, State Key Laboratory of Surface Physics, Fudan University, Shanghai 200433, China**

^5^**Shanghai Research Center for Quantum Sciences, Shanghai 201315, China**

**The PDF file includes:**

Figure S1 to S5

Note 1 and Note 2


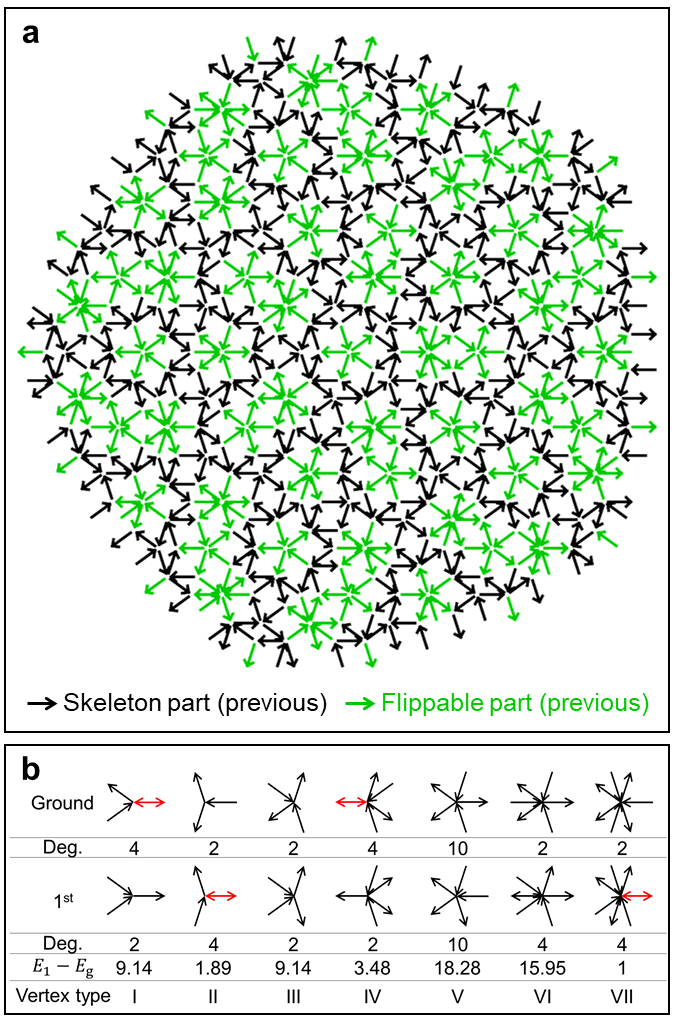


**Figure S1.** The candidate ground state for Type-I system proposed by Shi et al.^34^ (**a**) The spin configuration constructed by the elaborated logical steps under the consideration of interaction between nearest neighboring vertices. To distinguish from the skeleton-flippable configuration newly proposed through this study, the skeleton and flippable parts proposed by Shi et al. shown in (a) are marked with the “previous”. (**b**). The lowest (Ground) and second lowest (1^st^) energy configuration for each of seven different types of unit vertices in isolation. “Deg.” indicates the number of degenerate states. The energy difference between the lowest and second lowest energy levels has been normalized by that of Type-VII vertex. The figure in (b) is referred from the paper by Shi et al. The red double-headed arrows indicate the frustrated magnetic moments for each case.

**
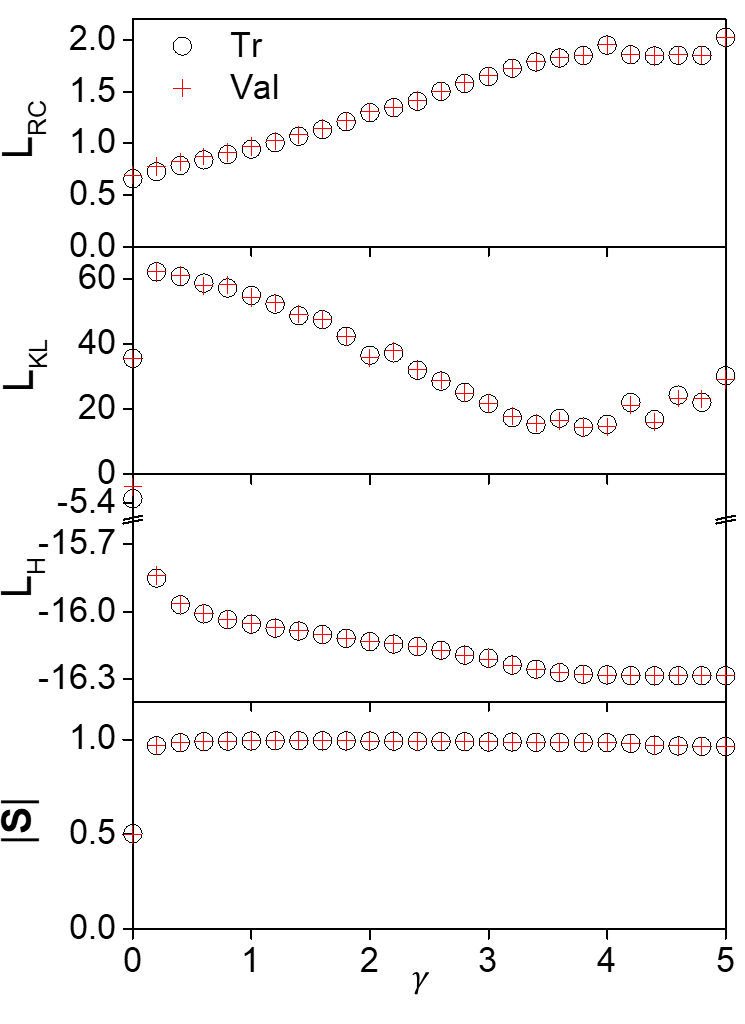
**

**Figure S2.** The behavior of several quantities from a training process of E-VAE model. Graphs for the reconstruction loss ($L_{\mathrm{RC}}$), KL loss ($L_{\mathrm{KL}}$), Hamiltonian loss ($L_{\mathcal{H}}$), and average of the amplitude values ​​of all components in generated output data ($\left| \boldsymbol{S} \right|$) are shown. Tr and Val indicate the results from training and validation datasets, respectively.

**Note 1.**

We follow several quantities ($L_{\mathrm{RC}}$, $L_{\mathrm{KL}}$, $L_{\mathcal{H}}$, and $\left| \boldsymbol{S} \right|$) during a training process of E-VAE model as shown in Fig. S2. (Detailed discussions for the purposes of $L_{\mathrm{RC}}$, $L_{\mathrm{KL}}$, and $L_{\mathcal{H}}$ loss terms are given in Methods section) As $\gamma$ increases, $L_{\mathrm{RC}}$ gradually increases and then saturates when $\gamma\sim4$. Considering the goal of $L_{\mathrm{RC}}$ term is to generate the output data identical to input data, increasing $L_{\mathrm{RC}}$ indicates that the trained E-VAE model can generate different spin configurations from the metastable spin states in our training dataset (input) obtained from simulated annealing processes. $L_{\mathrm{KL}}$ exhibits a behavior almost opposite to that of $L_{\mathrm{RC}}$ until $\gamma$ reaches about 4, and thereafter exhibits non-monotonic behavior. $L_{\mathcal{H}}$ decreases and then saturates as $\gamma$ increases, which means that the main purpose of E-VAE model to reduce the energy of output spin states is properly achieved. The $\left| \boldsymbol{S} \right|$ is immediately saturated with 1 when $\gamma$ is considered, and it indicates that each component in a generated spin state is close to either 1 or -1 integer. Thus, the output spin states generated from the trained E-VAE model can be considered as the spin states composed of $N$ normalized point dipoles as similar to the input data.


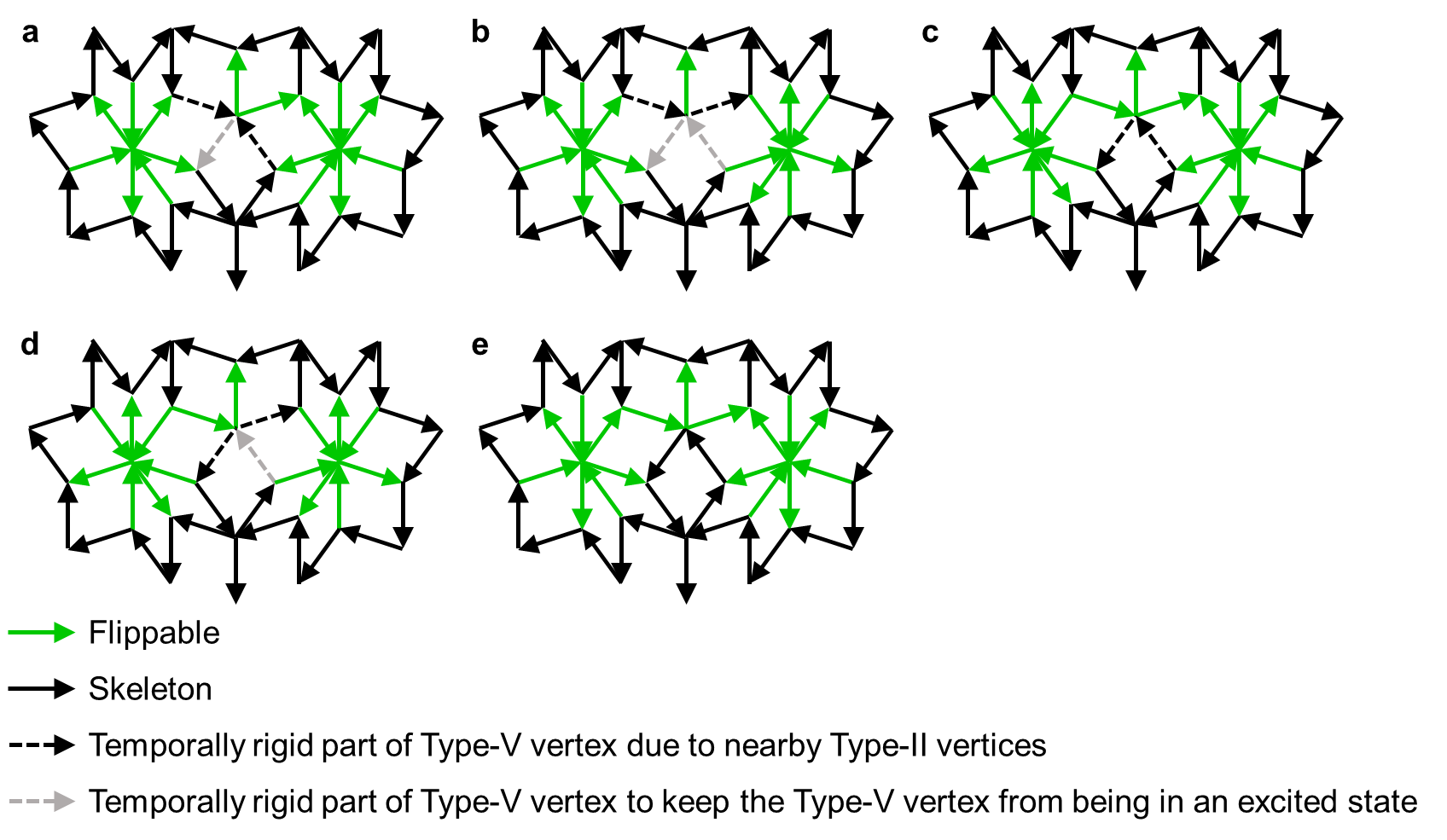


**Figure S3.** Newly revealed skeleton structure. (**a**) A sample spin configuration of a group of three joined flippable vertices (one Type-V vertex and two Type-VII vertices) to be considered initially. (**b-d**) Comparing with (a), the spin configurations (b) when the right Type-VII vertex is flipped, (c) when the left Type-VII vertex is flipped, and (d) when two Type-VII vertices are flipped simultaneously. (**e**) Our new skeleton-flippable configuration. Black and gray dashed arrows are the results of rigid part analysis performed on each of them.


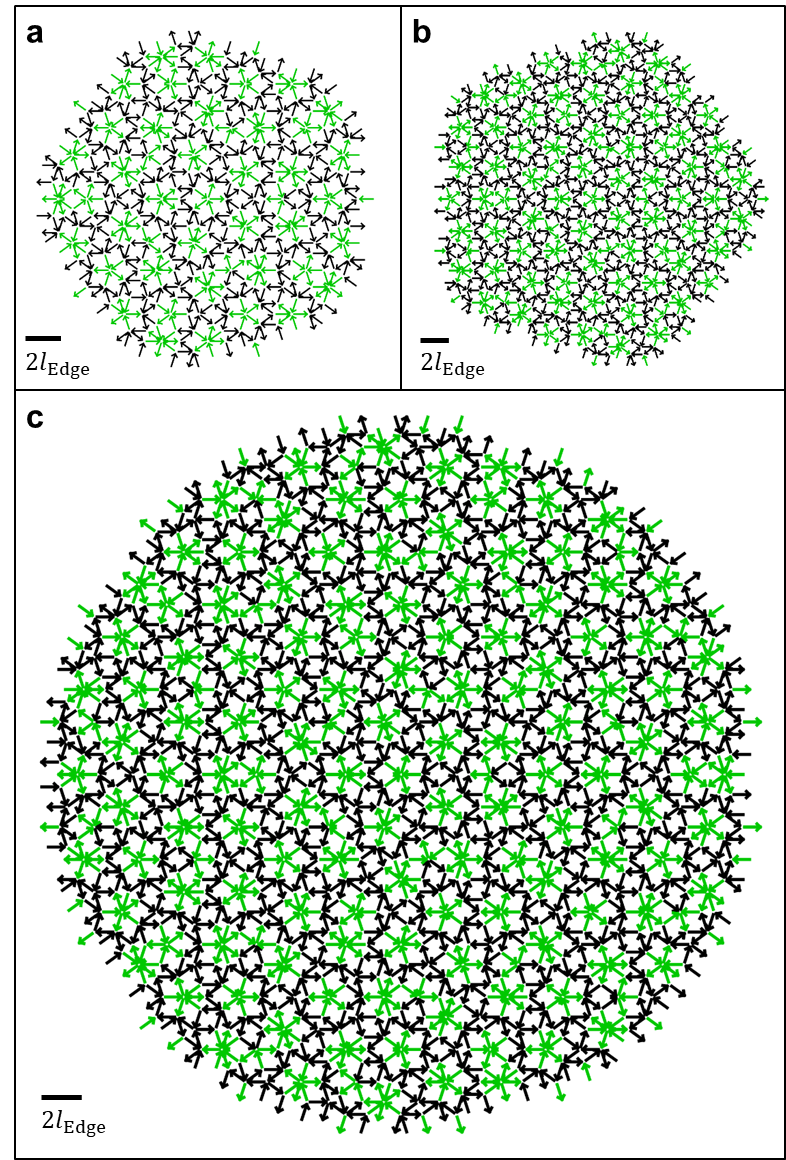


**Figure S4.** Generated spin states from the trained E-VAE models for Type-II systems of different sizes. Spin configurations formed on Type-II systems composed of (**a**) $N=640$, (**b**) $1195$, and (**c**) $2150$ spins. The black and green vectors indicate the skeleton and flippable parts for each system, respectively. All spin configurations are obtained from the trained E-VAE models for each of the systems. The scale bars represent twice the length of a single edge.


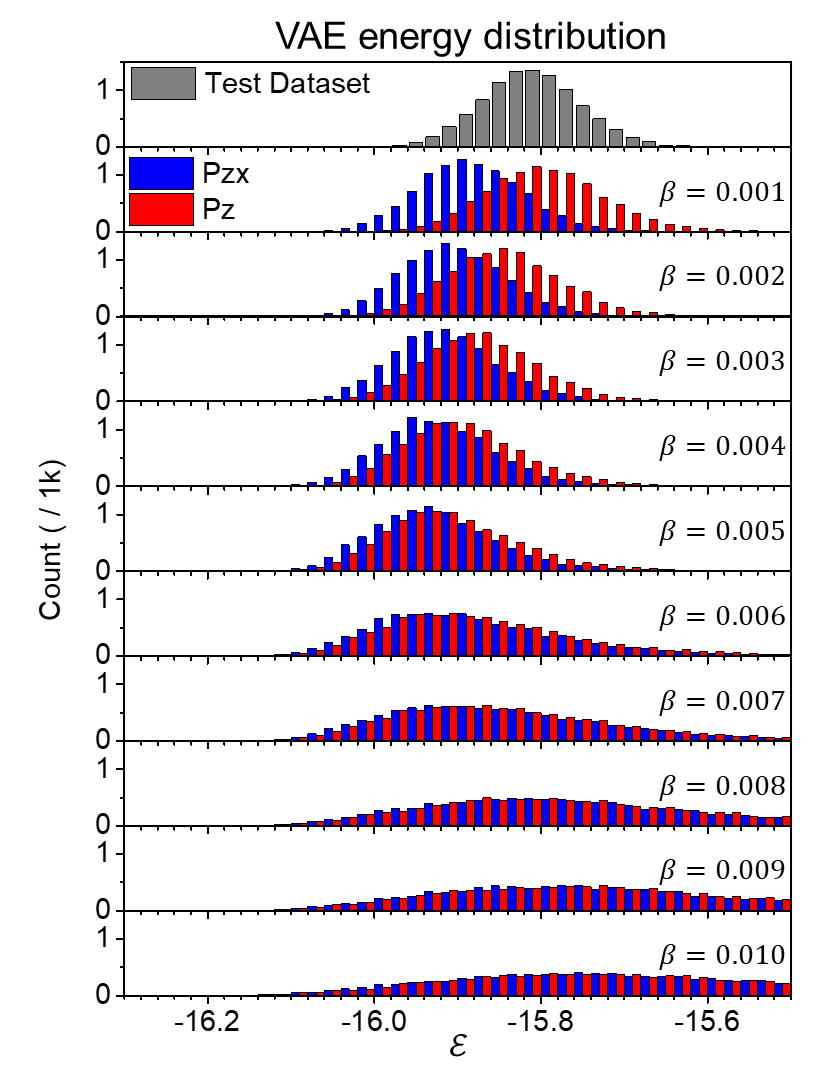


**Figure S5.** Determining the proper $\beta$ value using the results of VAE training. Energy distributions of the spin states generated from trained VAE models ($\gamma=0$). Pzx and Pz indicate the energy distributions for the spin states generated by the sampling from the $P(z|x)$ and $P(z)$, respectively.

**Note 2.**

The goal of conventional VAE model ($\gamma=0$ in $L_{\mathrm{Total}}=L_{\mathrm{RC}}+\beta L_{\mathrm{KL}}+\gamma L_{\mathcal{H}}$) is to be a good generator, and being a good generator means being able to generate new plausible data on its own without any input data. To achieve this goal, in usual VAE studies, they control the $\beta$ value to make the feature distributions of training dataset (a set of normal distributions constructed by the $\boldsymbol{\mu}$ and $\boldsymbol{\sigma}$ extracted from input data, $P(z|x)$) close to the standard normal distribution ($P(z)$); actually, this is the main purpose to consider $L_{\mathrm{KL}}$ term in the training process. Thus, it is believed that a well-trained VAE model with a proper $\beta$ value can generate new plausible data by sampling from the standard normal distribution without input data.

In this study, to determine the proper $\beta$ value, we train several VAE models with different $\beta$ values, and investigate the energy distributions of the spin states generated by the sampling from the $P(z|x)$ and $P(z)$ as shown in Fig. S5. Through a qualitative analysis, we speculate that $\beta=0.005$ is the best condition because the energy distributions from the $P(z|x)$ and $P(z)$ are similar each other. In addition, the peak of $P(z)$ distribution for that case is the lowest among all cases shown in the Fig. S5; it means that output data sampled from the standard normal distribution can be most energetically stable on average when $\beta=0.005$. Consequently, we set the $\beta$ to 0.005 and fix it for all E-VAE models shown in this study.
